# Resilience trajectories and links with childhood maltreatment in adolescence: a latent growth modeling approach

**DOI:** 10.1186/s13034-023-00558-2

**Published:** 2023-01-19

**Authors:** Agniete Kairyte, Inga Truskauskaite, Ieva Daniunaite, Odeta Gelezelyte, Paulina Zelviene

**Affiliations:** Center for Psychotraumatology, Institute of Psychology, Faculty of Philosophy, Vilnius University, M. K. Ciurlionio str. 29, Vilnius, 01300 Lithuania

**Keywords:** Resilience, Child maltreatment, Adolescence, Longitudinal study

## Abstract

**Background:**

The current definitions of resilience can be addressed as a process, an outcome, or a trait. Empirical studies should be carried out to determine the most appropriate definition for it. Therefore, the main aim of the current study was to investigate changes in adolescents’ resilience over two years and explore the links between resilience and different forms of child maltreatment.

**Methods:**

The three-wave longitudinal study “Stress and resilience in adolescence” (STAR-A) sample was comprised of a general school-based sample of Lithuanian adolescents [baseline *N* = 1295, 56.7% females; *M*(*SD*)_age_ = 14.24 (1.26)]. Resilience was measured using the 14-item Resilience Scale (RS-14), lifetime exposure to maltreatment was measured at wave 1 using a questionnaire developed by the Norwegian Center for Violence and Traumatic Stress Studies (NKVTS), risk of psychopathology—using the Strengths and Difficulties Questionnaire (SDQ). The changes in resilience scores over the period of two years were investigated using the latent growth modeling approach.

**Results:**

The analyses revealed two classes of resilience—stable higher and stable lower. We found that experience of at least one form of abuse was significantly more prevalent in the lower resilience group in comparison to the higher resilience group. Also, adolescents with lower resilience had a higher probability of psychopathology.

**Conclusions:**

This study provided meaningful insights into the stability of resilience over time in adolescence and its relation to various types of child maltreatment. Experiences of maltreatment, as well as risk for psychopathology, were linked to lower resilience in adolescence.

## Background

Resilience is a widely discussed yet complex psychological construct that includes biological, psychological, social, and cultural factors that interact with one another [1]. Several theoretical perspectives on resilience mostly debate whether resilience is a state or a trait. The theoretical concept that describes it as a state uses a perspective of resilience as a dynamic process encompassing positive adaptation within the context of significant adversity [2]. Within this perspective, what comes is that resilience could be enhanced as a capacity to handle upcoming adversities in life [3]. The other side of the debate takes an approach of resilience as a stable trajectory of functioning after a highly aversive event(s) [4, 5], and this hypothesis also has empirical evidence [6]. Much empirical evidence approves resilience-related environmental factors in both dynamic and stable trajectory perspectives, for example, the importance of specific family environment [7], relationships, gender, age, and education level [8] factors. Yet little is known about resilience in adolescence, considering that adolescence, per se, is a very dynamic developmental stage. However, there is an agreement between different perspectives on resilience that research in the context of experiences of aversive life events might help us better understand the resilience construct [1].

Adverse experiences in early childhood have been found to have a significant effect on a child’s psychosocial functioning and individual’s life at a later age. According to the World Health Organization (WHO), child maltreatment involves physical, sexual, psychological/emotional violence and neglect [9]. Different epidemiological studies provided reasonable evidence of the high prevalence of child abuse experiences across countries, giving the numbers around 50–83% [10–13]. Studies documented that child maltreatment is associated with various negative consequences, such as psychosocial problems [13], stress-related disorders [14], lower academic achievement [15, 16], and more frequent serious problems related to physical health in adulthood [17]. The disquieting data became an urgent impetus for preventing adverse childhood experiences and encouraging coping [9]. In this context, resilience has become an important research topic [1, 3].

In terms of resilience and experienced maltreatment, previous studies have found that overall less violence exposure [18], as well as decreased likelihood for traumatization [19], were associated with higher resilience. Also, studies showed that maltreatment experiences in childhood play an important role in the development of resilience [19]. In high-risk maltreated child groups, 20–57% of participants were identified as resilient [18, 20–22]. Nevertheless, not many studies have explored different types of maltreatment in the context of resilience. Thus, there is still a lack of understanding of whether specific experiences may be associated with lower or higher resilience. The effects of adversities on resilience is an ongoing question since longitudinal studies are challenging to perform. However, such studies can help to improve our knowledge of whether and how different types of maltreatment experiences affect the manifestation of resilience over the life course. Studies in this area may provide evidence-based knowledge on what factors are relevant for supporting resilience after potentially traumatic experiences. Such studies can also contribute to understanding whether resilience can be defined as a stable trait or a process. Therefore, the main aim of the current study was to investigate changes in adolescents’ resilience over two years and explore the links between resilience and different forms of child maltreatment.

## Methods

### Participants and procedure

The current study uses data from the three waves (W1, W2, and W3) of a larger and currently ongoing longitudinal research project “Stress and Resilience in Adolescence” (STAR-A) coordinated by the researchers from the Center for Psychotraumatology at Vilnius University, Lithuania. The initial STAR-A design was developed in collaboration with the Norwegian Center for Violence and Traumatic Stress Studies (NKVTS). The study aims to explore trauma and exposure to childhood abuse, traumatic stress reactions, and resilience in a general representative sample of adolescents.

The research project STAR-A was approved by the Ethics Committee for Psychological Research at Vilnius University. We used the quota sampling method in this study. The official invitation letters were sent to the public schools in different regions of Lithuania; 15 of them agreed to participate in the study. Inclusion criteria for the participants were: (1) age between 12 and 16 years old; (2) provided information about their age and sex. Prior to the data collection, written informed consent for participation in the longitudinal research project from at least one parent was obtained. More than half of parents agreed to their children’s participation in the study (56.8%), 14.9% declined the invitation, and 28.3% did not respond.

The data for W1 were collected in March-June 2019 using the paper–pencil method. With the assistance of a trained researcher, adolescents were provided with informed participation consent and self-report questionnaires. The data for W2 were collected in September–October 2020. Due to the COVID-19 (SARS-CoV-2) outbreak in Lithuania at that time, live contacts in schools were very limited. Therefore, an online data collection strategy was used. Data collection took place during the online lessons, and the assistance of a trained researcher was provided during an online meeting. Data collection for W3 took place in March-June 2021. Due to the COVID-19 lockdown measures in Lithuania, data was collected using an online strategy, using the same procedures as during W2 data collection. More information about the procedures and findings of the project can be found in previous publications [13, 23–25].

In total, 1299 adolescents participated in W1, but four did not meet the inclusion criteria, which made the final sample of 1295 adolescents. The final W1 sample comprised 56.7% (*n* = 734) females, and the mean age of participants was 14.24 (*SD* = 1.26, range 12–16) years. In total, 329 adolescents from 7 schools participated in W2, 57.4% (*n* = 189) were females, and the mean age was 15.35 (*SD* = 1.53, range 13–17). The sample of W3 comprised 849 adolescents, 58.5% (*n* = 497) females, and the mean age was 16.15 (*SD* = 1.35, range 13–18) years. Demographic information collected at W1 was used in the following data analysis.

The data collected during W1 revealed that 40.1% of the sample reported having financial difficulties in the family. Most participants’ mothers and fathers were employed (89.0% and 88.8%, respectively). In total, 39.5% of participants reported both parents’ university or college education, and almost one-third (29.9%) reported this level of education of one parent. One in five participants (21.9%) reported not knowing about their parents’ level of education (see Table 1).

**Table 1:** Demographic characteristics of study sample (*N* = 1295)

Variable	Total sample *N* = 1295	Lower resilience *n* = 113	Higher resilience *n* = 1183	χ^2^ (*df*)	*p*
	*n* (%)	*n* (%)	*n* (%)		
Sex					
Female	734 (56.7)	66 (58.9)	668 (56.5)	0.25 (1)	0.615
Male	561 (43.3)	46 (41.1)	515 (43.5)		
Age *M* (*SD*)	14.24 (1.26)	14.25 (1.21)	14.24 (1.27)	*t*(1293) = 0.86	0.931
Financial difficulties in the family^a^					
Yes	519 (40.1)	61 (55.0)	458 (38.8)	11.04 (1)	< .001
No	773 (59.7)	50 (45.0)	723 (61.2)		
Mother employed^a^					
Yes	1153 (89.0)	97 (87.4)	1056 (89.5)	6.32 (2)	0.043
No	123 (9.5)	10 (9.0)	113 (9.6)		
Not known	15 (1.2)	4 (3.6)	11 (0.9)		
Father employed^a^					
Yes	1150 (88.8)	96 (85.7)	1054 (89.5)	1.51 (2)	0.470
No	63 (4.9)	7 (6.3)	56 (4.8)		
Not known	77 (5.9)	9 (8.0)	68 (5.8)		
University/college education of parents^a^					
Both	512 (39.5)	26 (23.2)	486 (41.3)	16.13 (3)	0.001
One	387 (29.9)	38 (33.9)	349 (29.6)		
No	107 (8.3)	11 (9.8)	96 (8.1)		
Not known	284 (21.9)	37 (33.0)	247 (21.0)		
SDQ subscales^a^ *M*(*SD*)					
Emotional problems	2.97 (2.42)	5.05 (2.71)	2.77 (2.29)	*t*(125.49) = 8.62	< 0.001
Conduct problems	2.66 (1.41)	3.55 (1.56)	2.57 (1.36)	*t*(126.51) = 6.38	< 0.001
Hyperactivity	4.20 (1.77)	5.15 (1.95)	4.11 (1.73)	*t*(1270) = 5.94	< 0.001
Peer problems	3.35 (1.41)	4.11 (1.68)	3.27 (1.36)	*t*(122.78) = 5.05	< 0.001
Prosocial behavior	7.23 (2.04)	6.19 (2.18)	7.33 (1.99)	*t*(1281) = − 5.32	< 0.001
SDQ psychopathology prediction^a^					
No indication	813 (65.7)	26 (24.5)	787 (69.5)	91.51(2)	< 0.001
Possible	197 (15.9)	31 (29.2)	166 (14.7)		
Probable	228 (18.4)	49 (46.2)	179 (15.8)		

^a^Variables with missing data, range 0.2–0.4%

*SDQ* strengths and difficulties questionnaire

### Measures

The Resilience scale (RS-14) [26] was used to measure general psychological resilience. RS-14 covers five essential characteristics of resilience: purpose, perseverance, self-reliance, equanimity, and existential aloneness [26]. The resilience scale consists of 14 items, with a 7-point Likert scale from “Strongly disagree” (= 1) to “Strongly agree” (= 7). The total score is calculated by adding all items and may range from 14 to 98. Higher scores indicate higher resilience. The analysis of psychometric properties of the RS-14 in the sample of Lithuanian adolescents demonstrated high reliability of the scale [13]. In the current study, McDonald’s omega for W1/W2/W3 was 0.89/0.90/0.91, respectively.

Lifetime exposure to maltreatment was measured using a questionnaire developed by the NKVTS [27]. The questionnaire comprises 37 items, covering six possible types of abuse [28]: neglect (6 items), psychological abuse (8 items), physical abuse (6 items), sexual abuse from an adult (6 items), sexual abuse from peers (6 items), online sexual abuse (5 items). Different format of responses for each form of abuse was used. In response to neglect items, a 5-point scale, ranging from “Very rarely” (= 0) to “Very often” (= 4), was used; if the participant answered at least one item in scores ≥ 2, neglect was considered as experienced. Experience of psychological, physical, and all types of sexual abuse was measured on a 4-point scale, with possible answers “Never” (= 0), “Once” (= 1), “Occasionally” (= 2), and “Often” (= 3). Exposure to psychological abuse was considered as experienced if the participant responded to at least one item ≥ 2. Exposure to physical or sexual abuse was considered as experienced if at least one item defining that type of abuse was measured as “Once”, “Occasionally” or “Often”. The detailed information on specific types of maltreatment included in the questionnaire was published previously [13]. In the current study, lifetime exposure to maltreatment was measured at W1.

The Strengths and Difficulties Questionnaire (SDQ) [29] was used to measure risk for psychopathology and the level of different psychosocial difficulties. We used the total scores of the overall questionnaire and separate subscales. Difficulties subscales include Emotional symptoms, Conduct problems, Peer problems, and Hyperactivity. Strengths were measured using the Prosocial behavior subscale. The SDQ comprises 25 items (5 per each subscale) and probable answers “Not true” (= 0), “Somewhat true” (= 1), and “Certainly true” (= 2). The total difficulties score was obtained by adding the scores of all items of Emotional symptoms, Conduct behavior, Peer problems, and Hyperactivity subscales and ranged from 0 to 40. Lithuanian norms of the SDQ were published previously [30, 31]. Scores ranging from 0 to 14 show no indication of psychopathology, scores from 15 to 17 show possible psychopathology, and a total score ≥ 18 indicates probable psychopathology. The data of W1 of SDQ was used in the current study; McDonald’s omega for W1 was 0.83.

### Data analysis

The current study aimed to investigate the change in the resilience of adolescents over the period of two years, explore the patterns of change in resilience, and test the links between maltreatment exposure and resilience.

First, we investigated the changes in resilience scores using the latent growth modeling approach [32]. In latent growth models, *intercept* refers to the measure’s score at the baseline, while the *slope* refers to the change in measured construct over the chosen period. For the model estimation, we fitted the linear growth model. We fixed all the factor loadings of the intercept at 1 and the first-factor loading of the slope at 0. The next two factor loadings of the slope were loaded in correspondence to the time distance between measurement points, that is, at @1.5 and @2. Thus, the slope represented the change over one year in the current model. When running the latent growth model, we controlled for possible effects of gender (0 = female, 1 = male) and age by regressing them on both intercept and slope. Total scores of the RS-14 items were used in the analysis.

To explore the patterns of change in resilience over time, we conducted a univariate latent class growth analysis [33]. We used several criteria to decide on the number of latent classes, namely, relatively lower scores of the Akaike Information Criterion (AIC) and Bayesian Information Criterion (BIC), a statistically significant *p*-value of the adjusted Lo, Mendell, and Rubin test, Entropy score equal or above 0.70, indicative of the accurate classification, and the proportion of the participants in the smallest class not lower than 5% of the sample [33]. After deciding on the final number of classes, we conducted a series of Chi-square tests to indicate whether the proportions of adolescents exposed versus not exposed to at least one type of maltreatment or the particular type of maltreatment differed within the resilience classes.

The latent growth and latent class growth analyses were conducted with Mplus 8.2. [34]. The model fit in all analyses was evaluated by using the Comparative Fit Index (CFI), the Tucker–Lewis Index (TLI), and the Root Mean Square Error of Approximation (RMSEA) [35]. In particular, CFI/TLI values higher than 0.90 indicated an acceptable fit, and values higher than 0.95 represented a very good fit; RMSEA values below 0.08 indicated an acceptable fit, and values less than 0.05 suggested a good fit. The full information maximum likelihood (FIML) algorithm was used for handling missing data.

## Results

### Patterns of resilience

The results indicated that the conditional linear growth model with gender and age added as control variables fitted data well [χ^2^ (3) = 7.93, *p* = 0.047, CFI/TLI = 0.978/0.934, RMSEA (90% CI) = 0.036 (0.003, 0.067)]. No change in resilience was observed (*M*_intercept_ = 4.51, *M*_slope_ = − 0.09, *p* = 0.654). The significant variance of the intercept (Var_intercept_ = 0.60, *p* = 0.003) and non-significant variance of the slope (Var_slope_ = 0.10, *p* = 0.091) indicated that the adolescents might differ in terms of the baseline levels but not the change rates in resilience. A significant gender effect on intercept was found (β = 0.074, *p* = 0.042), indicating that boys reported higher resilience in comparison to girls.

The latent class growth analysis results showed that the two classes solution best fitted the data (see Table 2). Most of the study participants (91.4%) reported stable, relatively high rates of resilience (*M*_intercept_ = 4.86, *M*_slope_ = − 0.21, *p* = 0.270). The remaining subsample of adolescents (8.6%) reported stable lower rates of resilience (*M*_intercept_ = 3.00, *M*_slope_ = 0.39, *p* = 0.182).

**Table 2:** Model fit indices of latent class growth analysis

Solution	Loglikelihood	AIC	BIC	Entropy	LMR-A *p*-value	Smallest class count (%)
1 Class	− 3315.37	6654.73	6716.74	–	–	–
2 Classes	− 3271.84	6573.68	6651.18	0.78	0.030	8.6
3 Classes	− 3248.07	6532.13	6625.14	0.84	0.484	1.8

*AIC* Akaike information criterion, *BIC* Bayesian information criterion, *LMR-A* Lo-Mendell-Rubin adjusted likelihood ratio test (LMR-A)

The best-fitting solution is in bold

### Links between child maltreatment and resilience

Overall, 71.2% (*n* = 922) of adolescents reported at least one type of maltreatment. Almost half of the sample (*n* = 609; 47%) experienced at least one form of verbal abuse, 34.7% (*n* = 449) at least one form of physical abuse, 31.7% (*n* = 411) internet sexual abuse, 9.8% (*n* = 127) adult sexual abuse, 17.1% (*n* = 221) peer sexual abuse and 22.7% (*n* = 294) at least one form of neglect. Chi-square analyses were used to compare exposure to abuse in lower and higher resilience groups. Results revealed that more adolescents exposed to at least one form of neglect, verbal abuse, physical abuse, or internet sexual abuse were in the lower resilience group compared to those who had not been exposed to these forms of violence (Table 3).

**Table 3:** The proportions of participants in resilience groups within exposure to violence groups (*N* = 1295)

Types of maltreatment	Total sample (*N *= 1295)	Lower resilience (*n* = 112)	Higher resilience (*n* = 1183)	*χ*^*2*^ (1)	*p*
	*n *(%)	*n *(%) [Adj. Res]	*n *(%) [Adj. Res]		
Neglect	294 (22.7)	54 (48.2) [− 6.7]	240 (20.3) [6.7]	45.47	< 0.001
Psychological abuse	609 (47.0.)	81 (72.3) [− 5.6]	528 (44.7) [5.6]	31.40	< 0.001
Physical abuse	449 (34.7)	60 (53.6) [− 4.4]	389 (32.9) [4.4]	19.27	< 0.001
Internet sexual abuse	441 (31.7)	50 (44.6) [− 3.1]	361 (30.5) [3.1]	9.39	.002
Adult sexual abuse	127 (9.8)	11 (9.8) [0.0]	116 (9.8) [0.0]	0.00	1.000
Peer sexual abuse	221 (17.1)	25 (22.3) [− 1.5]	196 (16.6) [1.5]	2.38	0.123
At least one form of abuse	922 (71.2)	100 (89.3) [− 4.4]	822 (69.5) [4.4]	19.56	< 0.001

*Adj. Res* Adjusted standardized residuals

### Links between psychosocial functioning and resilience

Statistically significant differences between lower and higher resilience groups were revealed in all subscales of SDQ: emotional difficulties, conduct, hyperactivity, peer problems, and prosocial behavior. The scores in all subscales that define problems were higher in the lower resilience group. The higher scores on the prosocial behavior scale were found in the higher resilience group (see Table 1). Three groups of no indication of psychopathology (65.7%), possible (15.9%), and probable (18.4%) psychopathology, using SDQ scoring recommendations, were revealed. We found statistically significant differences between lower and higher resilience groups. Using Bonferroni-corrected *p*-value 0.05/3 = 0.017, we found that in comparison to ‘no indication of psychopathology’ vs. ‘possible’ and ‘no indication of psychopathology’ vs. ‘probable’, there were statistically significant higher proportions of ‘possible’ and ‘probable’ psychopathology in lower resilience group, than in higher resilience group (χ^2^ (1) = 46.81; *p* < 0.001, and χ^2^ (1) = 89.13; *p* < 0.001, respectively).

Moreover, Chi-square analyses revealed that the experience of financial difficulties in the family is more common in the lower resilience than in the higher resilience group (*p* < 0.001) (see Table 1). Also, differences were found in mother employment status and parents’ education variables between lower and higher resilience groups. Post hoc testing was carried out for mothers’ employment and parents’ education variables after choosing the Bonferroni-corrected *p*-values: 0.05/3 = 0.017 and 0.05/4 = 0.013, respectively. Significant differences were found only for some aspects of parents’ education. Adolescents whose both parents had a university or college education were less likely to be in the lower resilience group in comparison to those whose one parent had a university or college education (χ^2^ (1) = 7.49; *p* = 0.006), as well as those who do not know about their parent education (χ^2^ (1) = 15.84; *p* < 0.001) (Table 1).

## Discussion

This study enlarges evidence-based knowledge on resilience in adolescence. The current study aimed to explore resilience in adolescence from a longitudinal perspective and evaluate resilience in relation to different forms of child maltreatment. In a general sample of Lithuanian adolescents, two groups of stable lower and stable higher resilience have been identified within 8.6% and 91.4% of participants, respectively. The group of stable higher resilience is quite large compared to previous findings that reported around 20–57% of resilient adolescents [18, 20–22]. However, most previous studies included only adolescents exposed to maltreatment or other adverse experiences. Our study sample comprised adolescents from the general population and included both exposed and non-exposed to maltreatment. These findings reflecting the majority of the sample retaining higher resilience are encouraging.

Our results confirm the understanding of resilience as a stable trait. As the second and third waves of this study have been conducted during the COVID-19 pandemic, it is an important insight that resilience remains rather stable even during times of increased global burden on mental health [36]. Keeping in mind that adolescence is a developmental period characterized by the development of personality traits, it is necessary to understand that factors such as experienced maltreatment might influence the development of resilience. This study’s results align with previous studies, which found that experience of maltreatment is highly related to lower resilience [18, 19]. Our longitudinal data contribute to the evidence that experienced maltreatment might be a significant risk factor that links to lower resilience. Moreover, previous longitudinal studies have found that survivors of abuse are at higher risk of being re-victimized [37]. In these cases, continuous experiences of abuse may contribute to stable low resilience as a risk factor. Although maltreatment was measured only at the W1 time, we could not check these associations. Furthermore, meta-analyses found that higher levels of resilience are related to reduced risk for future traumatization [19]. Thus, the level of exposure to maltreatment and resilience might have reciprocal effects. However, regarding the discussion of whether resilience is dynamic or stable, it is necessary to address the methods used in this study. We used the 14-Item Resilience Scale (RS-14) [26]. The RS-14 was developed based on the theoretical assumption that resilience is a trait. Thus, it is important to emphasize that the results of this study are based on methodology, and there is a probability that the results are, at some level, biased.

The current study finds several types of maltreatment as risk factors for lower resilience. Adolescents exposed to at least one type of neglect, psychological, physical, or internet sexual abuse, were more likely to be in the lower resilience group. The proportions of adolescents who had experienced peer or adult sexual abuse did not differ between lower and higher resilience groups. However, Yoon et al. [20] did not find differences between exposure to different types of maltreatment and resilience. However, younger children (3–5 years) involved with the child welfare system participated in the aforementioned study. In samples at higher risk for psychosocial problems, multiple maltreatment types often co-occur, and detecting the differences might be more challenging. It is also important to note that groups of adolescents exposed to peer or adult sexual abuse were the smallest in our sample. It might as well be due to the fact that sexual abuse is the most intimate type of abuse that might be difficult to disclose, even on self-report questionnaires. Nevertheless, it would be important to continue similar studies with different types of maltreatment more prevalent in samples.

The results of this study also support previous findings regarding psychological difficulties and resilience [19, 38]. We found that adolescents at risk for psychopathology are more frequently characterized by stable lower resilience. Moreover, adolescents with stable lower resilience have higher levels of emotional, conduct, hyperactivity, and peer problems. Their prosocial behavior is lower than those adolescents, who have stable higher resilience. Furthermore, having psychopathology might lead to school absence, and during the data collection, we have not been able to collect data from these adolescents. However, going to school also could be a protective factor that enhances resilience to pathology, especially in adolescents with an abuse history in their families. In these cases, children who experienced maltreatment may consider school as a safe place from the perpetrators.

Furthermore, we identified sociodemographic factors that were related to lower and higher resilience. We found that males had higher rates of resilience than females, which is different compared to previous studies that found females to be more resilient [21] or had not found any sex differences [22]. This could be explained by differences in experienced maltreatment, as previous studies in Lithuania showed that females had been exposed to higher levels of physical and sexual abuse than males [13]. In addition, the results of the current study revealed that adolescents who live in families experiencing financial difficulties and whose only one parent has a college or university education or adolescents that do not know about their parents’ education were more likely to have stable lower resilience. These results support prior findings [20, 21]. Previous studies also found that child maltreatment is highly associated with lower levels of parental education and financial difficulties in families [39]. Our study raises assumptions that the development of stable lower resilience might be associated with this kind of family characteristics. However, further studies on how these factors are related from a longitudinal perspective are needed.

### Clinical implications

The longitudinal design of this study provided new and important findings for a long-lasting discussion regarding the theoretical approach of resilience as a construct—whether it is dynamic or stable. In our study, we found that resilience remains relatively stable over the period of three years. Risk factors for lower resilience were experienced neglect, psychological, physical abuse, or sexual abuse on the internet, as well as lower family income and lower education of parents. However, these risk factors might support the stability of low resilience. Thus, future longitudinal studies are in need. Early identification of risk groups and timely interventions to enhance resilience can be an important target for preventing future psychopathology and supporting healthy development.

### Limitations and future directions

To our knowledge, this is the first longitudinal study in Lithuania investigating the prevalence and associations between child maltreatment and patterns of resilience in adolescence. A large sample size and high response rate allowed us to explore different forms of child maltreatment and provide new knowledge about the prevalence of less frequent cases of abuse. The general population sample and the longitudinal study design provided a unique opportunity to investigate resilience trajectories and their relation to different maltreatment experiences over time. Although there are relevant strengths, the findings should be interpreted considering the limitations of the study. The two trajectories of resilience were identified in the general population and a relatively homogenous sample of Lithuanian adolescents. This result has generalizability limitations as different results might be found in diverse contexts and specific conditions [6]. The representativeness of the final sample was limited due to nearly half of the invited parents not giving informed consent for participation in the study. An ongoing COVID-19 pandemic also contributed to the high attrition rates of the sample. It is also necessary to address that in this study we measured exposure to abuse only in Wave 1. For future longitudinal research directions, it is essential to include this measure in the following waves to make sure that negative events experienced during the ongoing study would be taken into consideration as well. There is also a need to investigate other factors, including repeated maltreatment, that may contribute to the stability of resilience over time. Furthermore, it would be important to investigate links between child maltreatment and specific aspects of resilience.

## Conclusions

To sum up, the longitudinal study provided meaningful insights on the stability of resilience over time in adolescence and its relation to various types of child maltreatment. Results showed that at least one form of abuse was significantly more prevalent in the lower resilience group in comparison to the higher resilience group.

## Data Availability

The datasets of the current study are not publicly available due to ethical reasons but are available from the corresponding author upon reasonable request.

## Abbreviations

W1: First wave of the study
W2: Second wave of the study
W3: Third wave of the study
STAR-A: Stress and resilience in adolescence
NKVTS: Norwegian Center for Violence and Traumatic Stress Studies
RS-14: 14-Items resilience scale
SDQ: Strengths and difficulties questionnaire
AIC: Akaike information criterion
BIC: Bayesian information criterion
CFI: Comparative fit index
TLI: Tucker–Lewis index
RMSEA: Root Mean Square Error of Approximation
FIML: Full information maximum likelihood
